# Supplementary material for: Forecasting monthly residential natural gas demand in two cities of Turkey using just-in-time-learning modeling
Source: PLoS One. 2025 Jun 11;20(6):e0325538. doi: 10.1371/journal.pone.0325538 (PMC12157090; doi:10.1371/journal.pone.0325538)
Supplement: S1 Text — (DOCX) [file pone.0325538.s001.docx]

**S1 Text. Correlation of the Short-Term Effect in two Successive Months**

Estimating the functional form of $R_{y,m}$ in Equation 24 is not a straightforward task; in fact, linear combinations of lagged variable values are used in AR models, while a nonlinear kernel function is used in the GPR model (see below). Instead of estimating of $R_{y,m}$ directly, a method with minimum number of assumptions is employed to avoid both overcomplicating the correction procedure, and giving an advantage/disadvantage (bias) to the time series models over the JITL models (or vice versa). Hence, it is solely assumed that there exists (at least) lag-1 (one month) correlation between $D_{y,m}$ values for successive months, stemming from $R_{y,m}$ terms, i.e. $Corr(R_{y,m-1},R_{y,m})$. Noting that $R_{y,m}+e_{y,m}{=D_{y,m}-T}_{y,m}$, and the random error terms are independent, the lag-1 correlation can be estimated from $Corr \left( {\boldsymbol{D}_{m-1}-\boldsymbol{T}}_{m-1},{\boldsymbol{D}_{m}-\boldsymbol{T}}_{m} \right)$ using $\boldsymbol{D}_{m}={[D_{1,m} D_{2,m} \cdots D_{9,m}]}^{T}$ and $\boldsymbol{T}_{m}={[T_{1,m} T_{2,m} \cdots T_{9,m}]}^{T}$ for the *m*^th^ month. Indeed, the existence of this correlation is confirmed using the “clean” NGD data for Bursa and Kayseri; for instance, the correlation of the residual terms of the NGD data yielded a correlation coefficient of 0.34 with a *p*-value of 4.6×10^-3^.
