# Supplementary material for: Forecasting monthly residential natural gas demand in two cities of Turkey using just-in-time-learning modeling
Source: PLoS One. 2025 Jun 11;20(6):e0325538. doi: 10.1371/journal.pone.0325538 (PMC12157090; doi:10.1371/journal.pone.0325538)
Supplement: S2 Text — (DOCX) [file pone.0325538.s002.docx]

**S2 Text. Explanation of Optimization Function and its Constraints**

The first term in the minimization function (Equation 25 in the main text) represents the quality of the annual trend fits (square roots of the $R^{2}$ values) averaged over the number of optimized months, while the second term is the average absolute correlation of the residuals of the successive months, including one month earlier and later than the optimized season. The constraint in Equation 26 in the main text guarantees that the sum of the monthly NGDs during a season, e.g., July, August and September in a single year for Bursa, is equal to the sum of the measured monthly NGDs during the same period; the sum of the measured values, although monthly imprecise, actually represents the total consumption during the entire summer season. Finally, Equation 27 in the main text is the physical constraint of positive demand.
