# Supplementary material for: Forecasting monthly residential natural gas demand in two cities of Turkey using just-in-time-learning modeling
Source: PLoS One. 2025 Jun 11;20(6):e0325538. doi: 10.1371/journal.pone.0325538 (PMC12157090; doi:10.1371/journal.pone.0325538)
Supplement: S3 Text — (DOCX) [file pone.0325538.s003.docx]

**S3 Text. The rationale for using the specified ranges of window sizes for grid search**

The rationale for employing grid search for the window sizes over $W_{y}=\left\{ 2, 3, ...8 \right\}$ and $W_{m}=\{2, 3, \ldots6\}$ is listed as follows: (i) To include at least two months and two years in the local dataset, (ii) to include a large number of previous years, i.e., max($W_{y}$) = 8, to extract the yearly trend more accurately, (iii) to be able to model the sinosoidal-wave like behavior of NGD values between the months using a quadratic kernel; a quadratic polynomial is not expected to be a good fit for NGD values with a window size of $W_{m}>6$, since more than half of the whole cycle would be revealed in the local dataset.
