# Supplementary material for: Forecasting monthly residential natural gas demand in two cities of Turkey using just-in-time-learning modeling
Source: PLoS One. 2025 Jun 11;20(6):e0325538. doi: 10.1371/journal.pone.0325538 (PMC12157090; doi:10.1371/journal.pone.0325538)
Supplement: S4 Text — (DOCX) [file pone.0325538.s004.docx]

**S4 Text. Window size tuning for different months in JITL-GPR Method**

A similarity criterion is required to determine two RMSE surfaces, i.e., RMSE matrices, belonging to two successive months. First, $R_{i,j,k}$ is defined to be the RMSE corresponding to $(i,j)$ pair of ($W_{y}$, $W_{m}$) parameters with the month index *k*, and $\boldsymbol{R}_{k}=\left\{ R_{i,j,k}, i=1, 2,...7, j=1, \ldots5 \right\}\in\mathbb{R}^{35}$ (the numbers 7 and 5 are the cardinality of the set of $W_{y}$ and $W_{m}$ values, respectively). Then, the following function $I_{i,j,k}\in\left\{ 0,1 \right\}$ was defined; $H\left\{ \cdot\right\}$ is the heaviside function, and $quantile\left( \cdot, \alpha\right)$ determines the 100$\alpha$^th^ percentile of the given vector.

$I_{i,j,k}=H\left\{ quantile\left( \boldsymbol{R}_{k}, 0.40 \right)-R_{i,j,k} \right\}$ (Eq.1)

Therefore, $I_{i,j,k}=1$, if the RMSE value obtained for the corresponding parameter value is lower than the lower 40% percentile, and $I_{i,j,k}=0$, otherwise. The 40% threshold is based on a visual examination of the RMSE plots; the 50% percentile was deemed too high for allowing regions with high RMSE values to be included as acceptable, so it was slightly lowered. Finally, the $I_{i,j,k}$ values of subsequent months are multiplied and summed across the entire range of parameter values to ascertain the number of overlapping parameter couples with low RMSE values. For $k=1,2,\ldots12$,

$overlap\left( mod\left( k,12 \right),mod\left( k+1,12 \right) \right)=\sum_{i=1}^{7} \sum_{j=1}^{5} I_{i,j,mod\left( k,12 \right)}\times I_{i,j,mod\left( k+1,12 \right)},$ (Eq.2)

As illustrated in Figure S4, the overlap values (out of a maximum of $7\times5=35$ grid points) for all subsequent month pairs are displayed. Two clusters, consistent with the summer-beginning of winter classification, can be seen in Group II (June, July, August and September) and Group III (October, November and December). The pairwise month overlap values are high for the June-September season, with a decrease seen in September-October, which heralds the beginning of another group, a finding that is further confirmed by the high overlap seen for the October-December season. Unfortunately, high overlap values cannot be obtained for January-May season. While low RMSE values were obtained for January and February, when utilizing similar parameter sets, the RMSE profiles for March, April, and June seem to be distinctly different. Due to the limited number of historical observations, with each month having only five observations available for tuning the window size, it is not feasible to use separate groups for each of these months. Consequently, all months between January and May are grouped into a single category (Group I).


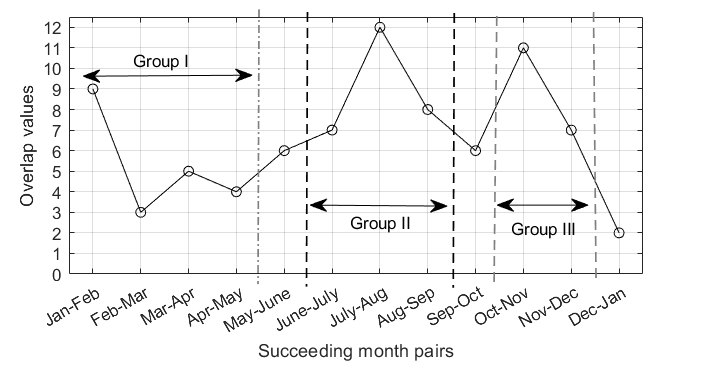


Figure S4. Overlap values of the succeeding months for Bursa dataset
