# Supplementary material for: Forecasting monthly residential natural gas demand in two cities of Turkey using just-in-time-learning modeling
Source: PLoS One. 2025 Jun 11;20(6):e0325538. doi: 10.1371/journal.pone.0325538 (PMC12157090; doi:10.1371/journal.pone.0325538)
Supplement: S5 Appendix — (DOCX) [file pone.0325538.s005.docx]

**S5 Appendix. Summary of NGDM studies in Turkey**

| **Reference** | **City ^*^** | **Prediction (Forecast) Horizon ^**^** | **Data Frequency** | **Database** | **Train/Test/Validation Ratio** | **Methods Used** | **Best Method** | **Performance Indicators Used** | **Temperature** | **Humidity** | **Wind** | **Public Holidays** | **Calendar Data (Weekday/Weekend)** |  |
| --- | --- | --- | --- | --- | --- | --- | --- | --- | --- | --- | --- | --- | --- | --- |
| [35] | I | D, H | 2008-2018 daily consumption | Istanbul Gas Distribution Company (IGDAS) | 75-15-10 | ANN | - | MSE | √ | X | X | √ | X |  |
| [48] | - | A | 2004-2012 annual consumption | Republic of Turkey Energy Market Regulatory Authority | 75-25-0 | GWO-FANGBM, GWO-GM, GM, ARIMA, LR | GWO-FANGBM | MAE, MAPE, RMSE | X | X | X | X | X |  |
| [57] | - | W | March 2020-March 2022 | Turkish Ministry of Energy and Natural Resources | 70-30-0 | ARIMA, NARNN, SVR, LSTM | LSTM | RMSE, MAE | X | X | X | X | X |  |
| [31] | AN | A, D, W | 2004-2013 daily consumption | Başkentgaz | 60-40-0 | LR, MARS, LASSO | MARS | MAPE, maxAPE, AAE, RMSE, $R^{2}$ | √ | √ | √ | X | √ |  |
| [45] | - | A | 2000 to 2019 annual consumption | BP Report (https://www.bp.com/) | 80-20-0 | Grey Prediction Models, GM Verhulst, Dynamic Grey Models | GM(1,5) | MAPE, RMSE, MSPE | X | X | X | X | X |  |
| [38] | - | M | 2010-2020 monthly consumption | jodidata.org | 73-27-0 | DEA, PSO, GSA and BSO Linear and Quadratic Models | PSO-Q | MAE, MAPE, RMS, MARNE, $R^{2}$ | √ | √ | √ | X | X |  |
| [32] | AN | D, W | 2004-2013 daily consumption | Başkentgaz | 60-40-0 | Ridge Regression, CMARS | CMARS | MAPE, AAE, RMSE, $R^{2}$ | √ | √ | √ | X | X |  |
| [33] | AN, B,  E, I | A, D,  M, W | 2002–2017 daily consumption | BOTAS Petroleum Pipeline Corporation | - | Fourier Series Expansion (FSE), Fourier Series Expansion with Temperature (FSET), Fourier Series Expansion with Temperature and Feedback (FSETF), AR Models | Fourier Series Expansion with Temperature and Feedback (FSETF) | MAPE, RMSE | √ | X | X | X | X |  |
| [34] | - | D | 2017–2019 daily consumption | Ministry of Energy and Natural Resources | 70-30-0 | ARIMAX, SARIMAX, ANN, NARX, LSTM, ARIMAX-ANN, SARIMAX-ANN, GA-ANN, PSO-ANN | SARIMAX-ANN | MAPE, RMSE, MSE, $R^{2}$ | X | X | X | X | √ |  |
| [55] | - | M | 2000-2018 monthly consumption | International Energy Agency | 75-25-0 | Seasonal Grey Forecasting Model, Adjusted Seasonal Grey Forecasting Model, SARIMA | Adjusted Seasonal Grey Forecasting Model | MAPE, MAE, RMSE, post-error ratio | X | X | X | X | X |  |
| [44] | - | A | 1998-2017 annual consumption | EnerData  https://www.enerdata.net/ | - | Artificial Bee Colony (ABC) Algorithm, Multiple Linear Regression | Artificial Bee Colony (ABC) Algorithm | MAPE | X | X | X | X | X |  |
| [54] | I | M | 2005–2015 monthly consumption | Istanbul Gas Distribution Company (IGDAS) | 90-10-0 | Multiple Linear Regression, ANN, SVM | SVM | MAPE, $R^{2}$ | √ | X | X | X | X |  |
| [30] | AN | D | 2009–2013 daily consumption | Başkentgaz | 80-20-0 | Linear Regression, NN, MARS, CMARS | CMARS | AAE, RMSE, MAPE | √ | √ | √ | X | √ |  |
| [41] | - | A | 1985-2000 annual consumption | Turkish Statistical Institute | - | Nonlinear Regression, Nonlinear Regression-based Breeder Genetic Algorithm, Nonlinear Regression-based Breeder Genetic Algorithm and Simulated Annealing | Nonlinear Regression-based Breeder Genetic Algorithm and Simulated Annealing | MAPE | X | X | X | X | X | |
| [29] | A | D | 2011-43 days 2012-366 days | Adapazarı Natural Gas Distribution Company (AGDAS) | - | Multiple Linear Regression | - | MAPE | √ | √ | X | √ | √ | |
| [56] | A | M | 2011–2014 monthly consumption | Adapazarı Natural Gas Distribution Company (AGDAS) | 75-25-0 | Holt-Winters Exponential Smoothing, ARIMA, SARIMA | ARIMA | MAPE, $R^{2}$, AIC, BIC | X | X | X | X | X | |
| [43] | - | A | 1985-2010 annual consumption | Ministry of Energy and Natural Resources | 65-35-0 | Hybrid Genetic Algorithm-Simulated Annealing (GA-SA) Algorithm, Multiple Linear Regression | GA-SA | MAPE, RE | X | X | X | X | X | |
| [1] | A | D | 2007-2011 daily consumption | Adapazarı Natural Gas Distribution Company (AGDAS) | 71-29-0 | SARIMAX, ANN-MLP, ANN-RBF, Multivariate OLS | ANN-MLP | MAPE, RMSE | √ | √ | √ | X | X | |
| [47] | - | A | 1987-2011 annual consumption | BOTAS Petroleum Pipeline Corporation | - | Linear and Logistic Models | Linear Models | RMSE, MAPE, $R^{2}$ | X | X | X | X | X | |
| [51] | I | D | 2004-2011 daily consumption | Istanbul Gas Distribution Company (IGDAS) | - | Analytical Model, Monte Carlo Simulation | - | RMSE | √ | √ | X | √ | √ | |
| [53] | - | W | 2002-2006 weekly consumption | BOTAS Petroleum Pipeline Corporation | 80-20-0 | ARIMA, ANN-MLP, ANN-RBF, ANNFIS | ANFIS | MAPE, RMSE | X | X | X | X | X | |
| [42] | - | A | 1984-2006 annual consumption | Ministry of Energy and Natural Resources | - | Linear and Quadratic Simulated Annealing | - | Absolute Value of Relative Errors | X | X | X | X | X | |
| [37] | I | M | 2004-2007 monthly consumption | Istanbul Gas Distribution Company (IGDAS) | 60-20-20 | ANN | - | ARE, $R^{2}$ | √ | X | X | X | X | |
| [40] | AN | A | 1991-2001 | BOTAS Petroleum Pipeline Corporation | - | Multiple Linear Regression | - | $R^{2}$ | √ | X | X | X | X | |
| [36] | I, AN,  B, E, K | M | 1996-2001 monthly consumption | EGO, IGDAS, BURSAGAZ, ESGAZ, IZGAZ | - | Autoregressive Time Series Models | - | MAPE, MAE, MSE, $R^{2}$ | √ | X | X | X | X | |

**^*^** A: Adapazarı, AN: Ankara, B: Bursa, E: Eskisehir, I: Istanbul, K: Kocaeli

**^**^** A: Annually, D: Daily, M: Monthly, Q: Quarterly, W: Week
