# Supplementary material for: Forecasting monthly residential natural gas demand in two cities of Turkey using just-in-time-learning modeling
Source: PLoS One. 2025 Jun 11;20(6):e0325538. doi: 10.1371/journal.pone.0325538 (PMC12157090; doi:10.1371/journal.pone.0325538)
Supplement: Attachment — Submitted filename: PONE-D-25-09289_Response-to-Reviewers.pdf [file pone.0325538.s007.pdf]

RESPONSE TO THE REVIEWER

1. ***Does the paper address the challenges of implementing JITL-GPR in real-world forecasting scenarios?***

   In light of your recommendation, the following sentences have been incorporated into the third paragraph of the conclusion (Lines 799-802):

   > "Despite the fact that the proposed approach is applicable to any monthly NGD data comprising 50 to 200 observations, it may encounter certain challenges when implemented in real-world forecasting. The method necessitates the aggregation of 'similar' months; however, this should be automated in future research to avoid selection bias."

2. ***Does the proposed JITL-GPR method provide improved forecasting accuracy compared to traditional models such as SARIMA and ETS?***

   Table 6 (higher test performances shown in bold) has been revised to include the performance metrics of the best statistical forecast model (i.e., TBATS) for both cities.

City	Bursa		Kayseri	
Model	JITL-GPR	TBATS	JITL-GPR	TBATS
$RMSE_{1\text{-}step}$ (msm^3)	13.23	9.02	6.407	5.221
$MAE_{1\text{-}step}$ (msm^3)	8.485	6.355	4.590	3.446
$MAPE_{1\text{-}step}$ (%)	12.20	11.08	16.34	13.19
$RMSE_{test}$ (msm^3)	**12.69**	14.86	**5.110**	6.786
MAE_{test} (msm^3)	**9.424**	9.666	**3.711**	5.094
$MAPE_{test}$ (%)	**13.51**	14.24	14.63	**13.26**
NGD in 2023 (msm^3)	816.8	816.8	477.6	477.6
NGD in 2023 prediction (msm^3)	**824.3**	779.3	**473.6**	451.0
Yearly PE in 2023	**0.90**	-4.58	**-0.83**	-5.55

   Figure 9 also demonstrates the superior performance of the proposed approach (i.e., JITL-GPR) over the other forecast models used for comparison in the present study.

3. ***To what extent do external factors (e.g., temperature, gas price, and number of subscribers) influence forecasting accuracy?***

   It is imperative to reiterate that the objective of this study is to construct an ex-ante predictive model, as opposed to an explanatory model, that can be utilized under realistic conditions. This entails using solely the knowledge that is available (or can be accessed) at the time of the forecasting; and one-year ahead temperature and gas prices cannot be known accurately at the time of forecasting. This is why we have deliberately avoided including these exogenous variables in forecasting models. In reference to your comment concerning the incorporation of the number of subscribers within the model, it is important to note that the proposed approach was also implemented using monthly natural gas consumption per subscriber; however, the findings did

not demonstrate any substantial improvement. Moreover, similar to temperature and price data, the implementation of such a model would necessitate the prediction of the number of subscribers in the forthcoming months, which would introduce additional uncertainty. Consequently, the present findings were reported solely.

We hope that the following information, which is already available in the first draft of the present study, addresses your concern regarding the inclusion of external factors such as temperature, weather, and gas price into the model:

- "This may, in fact, be due to the dominant factors at different forecast horizons; monthly to quarterly forecasts are mostly and almost equally affected by temperature and economy [2], and both variables are difficult to predict for monthly periods [4,5]." (Page 3)
- "One-day-ahead estimations of weather-related variables might be beneficial for short-term forecasting; nevertheless, forecasts of weather-related variables for more than a week would be less reliable, thereby diminishing the accuracy of exogenous variable models for monthly NGDP [15,49]." (Page 7)
- "However, the low-frequency change of exogenous variables, such as GDP, coupled with the challenging nature of accurately predicting variables like the inflation rate, poses a significant challenge in obtaining reliable monthly point NGDPs [50]." (Page 7)

Moreover, the following sentences have been incorporated into the conclusion to underscore the scope of the present study:

- "The present study aims to construct an ex-ante predictive model (rather than an explanatory model) that can be used under realistic conditions." (Lines 768-769)
- "The proposed approach has also been applied to monthly natural gas consumption per subscriber, but the results did not indicate a significant improvement. Furthermore, such a model would require the prediction of the number of subscribers in the coming months, which would introduce additional uncertainty. Therefore, only the current results were reported." (Lines 807-811)

4. ***Why are monthly forecasts less accurate compared to daily or yearly predictions?***

As previously stated in the response to Comment #3, the utilization of the actual values of weather-related variables in monthly predictions is not feasible, as they are known to be highly inaccurate. Conversely, hourly or daily weather-related predictions can attain a certain degree of accuracy, thereby facilitating the forecasting of natural gas consumption over these brief periods.

5. ***Can the JITL-GPR method be generalized to other cities or countries that rely on imported natural gas?***

We appreciate your inquiry regarding the generalizability of our methodology. The proposed approach was employed using NGD data of two cities in Turkey, which imports natural gas. There are no unique aspects of these cities; however, the prediction of NGD in them was requested by the NG distribution company (SOCAR) provided the data set used in our study.

Our methodology can be applied to any city or country with a moderate historical time series. To illustrate this point, we applied the methodology to Turkey's NGD data (2010-2020, 129 months) from https://www.kaggle.com/datasets/naturalgas/turkeys-monthly-natural-gas-data. The initial 108 months (2010-2018) were designated for training, while the final 21 months (2019-2020) served as the test data. The JITL-GPR method was implemented, with hyperparameters being adjusted on the training set, as outlined in our manuscript. Despite the selection of the SARIMA model, based on its minimized AIC, its performance on the test set was found to be unsatisfactory. Through a process of trial and error, the SARIMA model with the smallest RMSE on the test set was ultimately chosen. Notwithstanding this advantage, the JITL-GPR model demonstrated superior accuracy as evidenced in Figures 1 and 2. For instance, JITL-GPR accurately predicted the maximum NGD in January 2020, while SARIMA underpredicted it, thereby demonstrating JITL-GPR's superiority in handling extreme values.

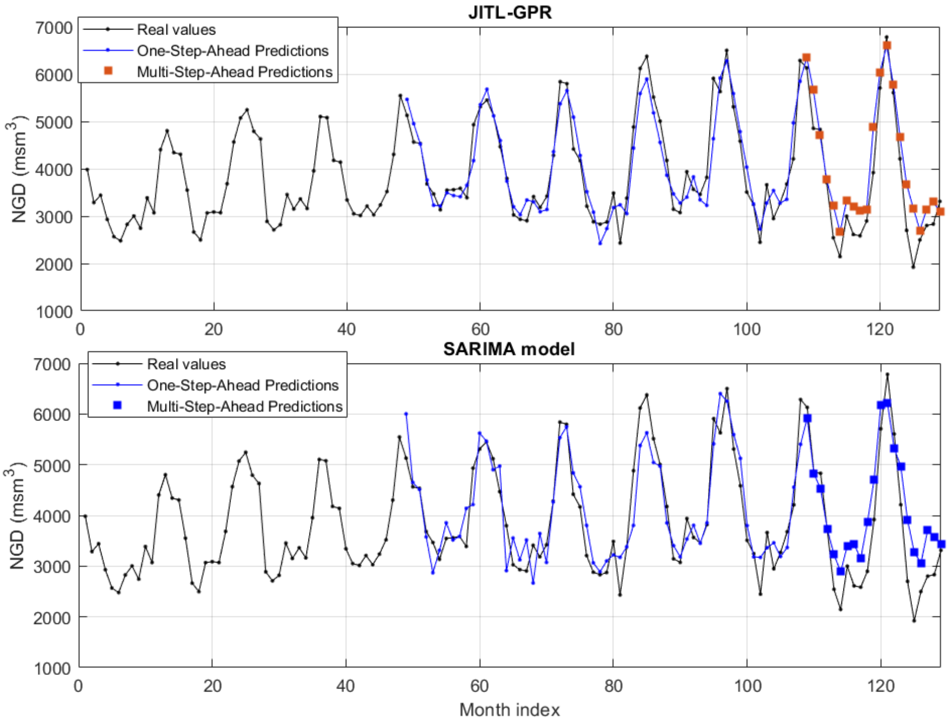

Figure 1. Real (red) and predicted (blue) NGD values of Turkey via (A) JITL-GPR, and (B) SARIMA models. Small and squared blue points correspond to one-step-ahead prediction in the training set, and multi-step-ahead predictions in the test set, respectively.

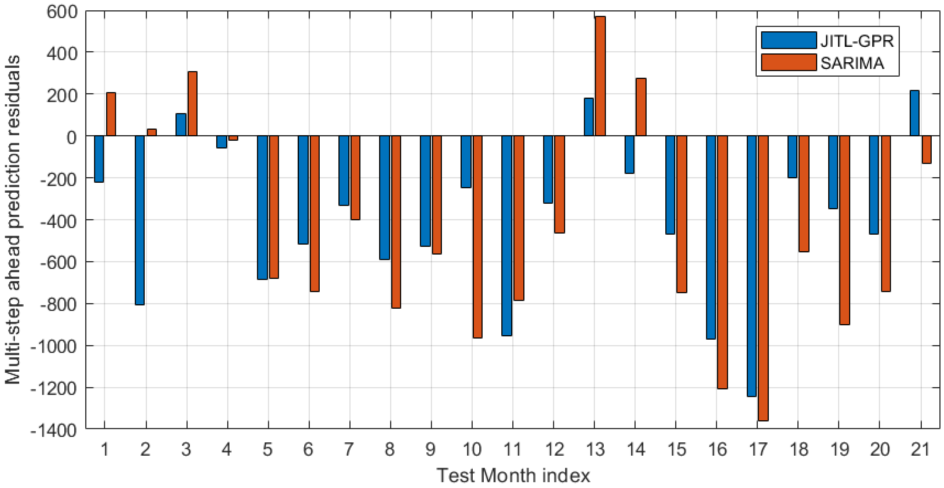

Figure 2. Multi-step ahead prediction residuals obtained from JITL-GPR (blue) and SARIMA (red) models.

As illustrated in Table 1, the JITL-GPR model exhibited a MAPE of approximately 15%, which is analogous to the results observed for Bursa and Kayseri in the manuscript, thus substantiating the reliability of the employed metrics for future assessments. In contrast, SARIMA exhibited a MAPE of 20% and a RMSE that was 25% worse than that of JITL-GPR, thereby underscoring the superior predictive accuracy of the former.

Table 1. A summary of prediction metrics via JITL-GPR and SARIMA models for NGD values of Turkey.

	Method	
**Metric**	**JITL-GPR**	**SARIMA**
**$RMSE_{1\text{-}step}$ (msm^3)**	385.4	461.0
**$MAPE_{1\text{-}step}$ (%)**	7.69	9.36
**$RMSE_{test}$ (msm^3)**	554.7	691.5
**$MAPE_{test}$ (%)**	15.6	20.6

For your information: The purpose of presenting these findings is to facilitate a more profound comprehension of the efficacy of the proposed approach. It should be noted that these findings have not been incorporated into the revised version of the manuscript.

6. ***Most of the figures are not clear, please.***

The images in the first draft have been replaced with their high-resolution versions.
