# Supplementary material for: Forecasting monthly residential natural gas demand in two cities of Turkey using just-in-time-learning modeling
Source: PLoS One. 2025 Jun 11;20(6):e0325538. doi: 10.1371/journal.pone.0325538 (PMC12157090; doi:10.1371/journal.pone.0325538)
Supplement: Attachment — Submitted filename: PONE-D-25-09289_Response-to-Reviewers-v2.pdf [file pone.0325538.s008.pdf]

RESPONSE TO THE REVIEWER

1. ***Does the paper address the challenges of implementing JITL-GPR in real-world forecasting scenarios?***

   In light of your recommendation, the following sentences have been incorporated into the third paragraph of the conclusion (Lines 852-859):

   > "Despite the fact that the proposed approach is applicable to any monthly NGD data comprising 50 to 200 observations, there is still room for improvement with regard to real-world forecasting. The proposed method for aggregation of months, which involves the partitioning of a single year into three periods (January-May, June-September, and October-December), and the proposed kernel function have demonstrated effectiveness in the tested datasets. However, there is potential for enhancement of both the aggregation process and the kernel function in the future, with the objective of aligning with the NGD values derived from a more extensive range of geographic and socioeconomic conditions."

2. ***Does the proposed JITL-GPR method provide improved forecasting accuracy compared to traditional models such as SARIMA and ETS?***

   Table 6 (higher test performances shown in bold) has been revised to include the performance metrics of the best statistical forecast model (i.e., TBATS) for both cities.

City	Bursa		Kayseri	
Model	JITL-GPR	TBATS	JITL-GPR	TBATS
$RMSE_{1\text{-}step}$ (msm^3)	13.23	9.02	6.407	5.221
$MAE_{1\text{-}step}$ (msm^3)	8.485	6.355	4.590	3.446
$MAPE_{1\text{-}step}$ (%)	12.20	11.08	16.34	13.19
$RMSE_{test}$ (msm^3)	**12.69**	14.86	**5.110**	6.786
MAE_{test} (msm^3)	**9.424**	9.666	**3.711**	5.094
$MAPE_{test}$ (%)	**13.51**	14.24	14.63	**13.26**
NGD in 2023 (msm^3)	816.8	816.8	477.6	477.6
NGD in 2023 prediction (msm^3)	**824.3**	779.3	**473.6**	451.0
Yearly PE in 2023	**0.90**	-4.58	**-0.83**	-5.55

   Figure 9 also demonstrates the superior performance of the proposed approach (i.e., JITL-GPR) over the other forecast models used for comparison in the present study.

3. ***To what extent do external factors (e.g., temperature, gas price, and number of subscribers) influence forecasting accuracy?***

   It is imperative to reiterate that the objective of this study is to construct an ex-ante predictive model, as opposed to an explanatory model, that can be utilized under realistic conditions. This entails using solely the knowledge that is available (or can be accessed) at the time of the forecasting; and one-year ahead temperature and gas prices cannot be known accurately at the

time of forecasting. This is why we have deliberately avoided including these exogenous variables in forecasting models. In reference to your comment concerning the incorporation of the number of subscribers within the model, it is important to note that the proposed approach was also implemented using monthly natural gas consumption per subscriber; however, the findings did not demonstrate any substantial improvement. Moreover, similar to temperature and price data, the implementation of such a model would necessitate the prediction of the number of subscribers in the forthcoming months, which would introduce additional uncertainty. Consequently, the present findings were reported solely.

We hope that the following information, which is already available in the first draft of the present study, addresses your concern regarding the inclusion of external factors such as temperature, weather, and gas price into the model:

- "This may, in fact, be due to the dominant factors at different forecast horizons; monthly to quarterly forecasts are mostly and almost equally affected by temperature and economy [2], and both variables are difficult to predict for monthly periods [4,5]." (Page 3)
- "One-day ahead estimations of variables related to weather might be helpful for short-term forecasting; however, forecasts of weather-related variables for longer than a week period would be less reliable, thereby decreasing the accuracy of exogenous variable models for monthly NGDP [15,49]." (Page 7)
- "However, the low-frequency change of exogenous variables, such as GDP, coupled with the challenging nature of accurately predicting variables like the inflation rate, poses a significant challenge in obtaining reliable monthly point NGDPs [50]." (Page 7)

Moreover, the following sentences have been incorporated into the conclusion to underscore the scope of the present study:

- "The present study aims to construct an ex-ante predictive model (rather than an explanatory model) that can be used under realistic conditions." (Lines 823-824)
- "The proposed approach was also applied to monthly natural gas consumption per subscriber, but the results did not indicate a significant improvement. Furthermore, such a model would require the prediction of the number of subscribers in the coming months, which would introduce additional uncertainty. Therefore, only the current results were reported." (Lines 864-867)

4. ***Why are monthly forecasts less accurate compared to daily or yearly predictions?***

As previously stated in the response to Comment #3, the utilization of the actual values of weather-related variables in monthly predictions is not feasible, as they are known to be highly inaccurate. Conversely, hourly or daily weather-related predictions can attain a certain degree of accuracy, thereby facilitating the forecasting of natural gas consumption over these brief periods.

5. ***Can the JITL-GPR method be generalized to other cities or countries that rely on imported natural gas?***

We appreciate your inquiry regarding the generalizability of our methodology. The proposed approach was employed using NGD data of two cities in Turkey, which imports natural gas. There are no unique aspects of these cities; however, the prediction of NGD in them was requested by the NG distribution company (SOCAR) provided the data set used in our study.

Our methodology can be applied to any city or country with a moderate historical time series. To illustrate this point, we applied the methodology to Turkey's NGD data (2010-2020, 129 months) from https://www.kaggle.com/datasets/naturalgas/turkeys-monthly-natural-gas-data. The initial 108 months (2010-2018) were designated for training, while the final 21 months (2019-2020) served as the test data. The JITL-GPR method was implemented, with hyperparameters being adjusted on the training set, as outlined in our manuscript. Despite the selection of the SARIMA model, based on its minimized AIC, its performance on the test set was found to be unsatisfactory. Through a process of trial and error, the SARIMA model with the smallest RMSE on the test set was ultimately chosen. Notwithstanding this advantage, the JITL-GPR model demonstrated superior accuracy as evidenced in Figures 1 and 2. For instance, JITL-GPR accurately predicted the maximum NGD in January 2020, while SARIMA underpredicted it, thereby demonstrating JITL-GPR's superiority in handling extreme values.

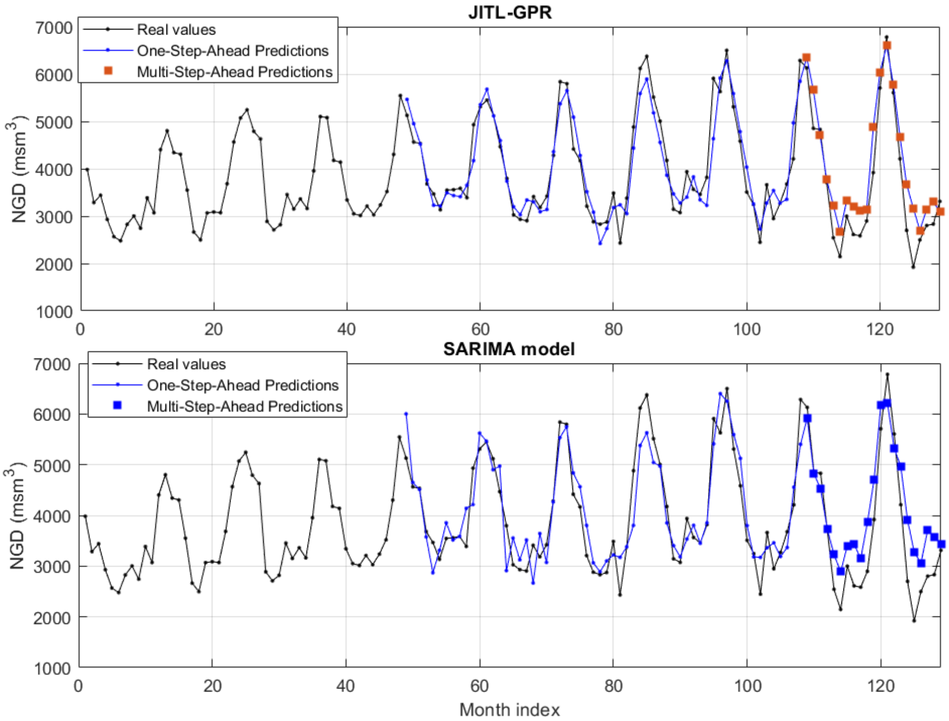

Figure 1. Real (red) and predicted (blue) NGD values of Turkey via (A) JITL-GPR, and (B) SARIMA models. Small and squared blue points correspond to one-step-ahead prediction in the training set, and multi-step-ahead predictions in the test set, respectively.

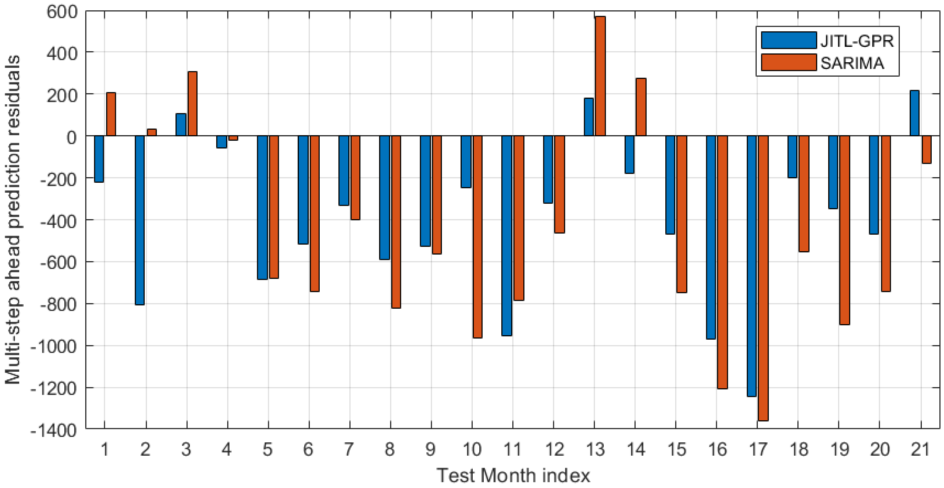

Figure 2. Multi-step ahead prediction residuals obtained from JITL-GPR (blue) and SARIMA (red) models.

As illustrated in Table 1, the JITL-GPR model exhibited a MAPE of approximately 15%, which is analogous to the results observed for Bursa and Kayseri in the manuscript, thus substantiating the reliability of the employed metrics for future assessments. In contrast, SARIMA exhibited a MAPE of 20% and a RMSE that was 25% worse than that of JITL-GPR, thereby underscoring the superior predictive accuracy of the former.

Table 1. A summary of prediction metrics via JITL-GPR and SARIMA models for NGD values of Turkey.

	Method	
**Metric**	**JITL-GPR**	**SARIMA**
**$RMSE_{1\text{-}step}$ (msm^3)**	385.4	461.0
**$MAPE_{1\text{-}step}$ (%)**	7.69	9.36
**$RMSE_{test}$ (msm^3)**	554.7	691.5
**$MAPE_{test}$ (%)**	15.6	20.6

For your information: The purpose of presenting these findings is to facilitate a more profound comprehension of the efficacy of the proposed approach. It should be noted that these findings have not been incorporated into the revised version of the manuscript.

6. ***Most of the figures are not clear, please.***

The images in the first draft have been replaced with their high-resolution versions.

RESPONSE TO THE ACADEMIC EDITOR

1. The authors did not include the results for the additional dataset in the manuscript, explaining it would make the paper overly long.

   We would like to express our gratitude to the academic editor for providing us with the opportunity to implement our proposed method on an additional set of data and present the results. In the latest version of the manuscript, we have incorporated a new section entitled “Application of JITL-GPR on an additional dataset of NGD in Turkey” (Pages 33-35), accompanied by Table 7 and Figure 12, which are situated just above the Conclusion. This section discusses the results pertaining to the new dataset. A comparison of the proposed method (JITL-GPR) with SARIMA has been made, and it has been demonstrated that the prediction accuracy of JITL-GPR exceeds that of SARIMA, even under conservative settings (please also see the next response).

2. They did not implement automated selection of similar months for local model training, though they acknowledged it as a promising future direction to mitigate "selection bias".

   We would like to express our sincere gratitude to the academic editor for bringing this matter to our attention. We believe that the phrase “selection bias” was either redundant or misleading. It could easily mislead the reader into thinking that our month grouping was biased. For example, potential readers may believe that months were grouped in a certain way to increase prediction accuracy, i.e., data leakage from test data. Indeed, it is not, and data leakage is an issue that we have deliberately avoided in the present study since the proposed method is planned to be used in real industry. We reviewed the month partitioning strategy (which is more statistical in nature, as opposed to the automated nature encountered more frequently in machine learning) in the original version of the manuscript (please refer to subsection *Window Size Tuning* and *Supporting Text S4*) for Bursa using only the training data. We proposed dividing a year into three groups: January-May, June-September, and October-December. Please be advised that the partitioning procedure is comparable to other NGDP studies, in which weekdays and weekends are treated separately, and a year is partitioned into four seasons. However, the partitioning in the present study is more finely tuned for NGDP habits. Therefore, the method we employed ensured the prevention of data leakage, even in the case of Bursa. Furthermore, we implemented the same partitioning strategy for Kayseri and reported the obtained result. In the latest version of the manuscript, we extended the same partitioning for a new dataset, and obtained the currently reported results (please see item [*i*] below). Therefore, there is no “selection bias” in our current methodology. Our selection method is based on a statistical treatment of training data from a single city that can be applied to data from another city or the entire country.

   The reason for using this term, which is not accurate, was based on a misinterpretation of the phrase “challenges” in the reviewer’s query, which read, “Does the paper address the challenges of implementing JITL-GPR in real-world forecasting scenarios?” In the present study, we did not utilize any simulation or “toy” data. The entire method we proposed was designed to address the challenges of a real-world forecasting scenario. However, to respond to the reviewers’ query using the original phrase, it is possible that we erroneously used the term “selection bias” as an example of “challenge.” Instead, we should have clarified that different partitioning methods (see item [*ii*] below) may be developed in the future to accommodate NGD data from various sources. To address this misunderstanding, we implemented the following changes:

i. We employed the same groupings, kernel parameter, and hyperparameter search ranges in the new dataset as we used for Bursa and Kayseri. As a result, we used the same hyperparameters for all three datasets. It should be noted that since the new dataset was not included in the previous version of our manuscript, the new results related to our proposed method (since it uses the same groups of months and other hyperparameters) are unbiased predictions. That is to say, the month partitionings are not changed for the new dataset to improve test prediction accuracy. This point is emphasized in the newly added section:

"To circumvent selection bias in the combination of similar months for the JITL-GPR method, the identical month groupings that were employed for Bursa and Kayseri (see Supporting Text S4) were utilized. Moreover, the identical hyperparameter search space and kernel function were employed to ascertain the hyperparameters with the minimal validation errors." (Page 34, Lines 791-795).

It should also be noted that the other prediction method (SARIMA), which was used for comparison, was indeed a biased model. It was selected to achieve the best possible test accuracy. However, our proposed method demonstrates superior prediction accuracy, as outlined in Table 7 on Page 34.

ii. We have removed the sections of the manuscript that may incorrectly suggest that our method of grouping months is biased. To address the aforementioned misunderstanding, we have revised the related section as follows:

"…there is still room for improvement with regard to real-world forecasting. The proposed method for aggregation of months, which involves the partitioning of a single year into three periods (January-May, June-September, and October-December), and the proposed kernel function have demonstrated effectiveness in the tested datasets. However, there is potential for enhancement of both the aggregation process and the kernel function in the future, with the objective of aligning with the NGD values derived from a more extensive range of geographic and socioeconomic conditions." (Pages 36-37, Lines 853-859).

As previously mentioned and included in the latest version of the manuscript, the proposed partitioning method and the kernel function are two parts of the algorithm that may be improved (or not) when NGD values from different regions are used, just like any method. While we strongly support the use of the same month groupings and kernel functions for datasets from other countries, we acknowledge the potential for further refinement, particularly for countries geographically distant from Turkey. Consequently, future enhancements will not entail the elimination of an existing "selection bias" in our method (which does not possess one), but rather the expansion of our scope to encompass a more extensive range of NGD characteristics. However, this is a separate topic that merits further research. It should include large and varied datasets of NGD time series, potentially covering different decades from various regions worldwide. Additionally, it should incorporate a range of search approaches. Finally, we would like to emphasize that the results in our manuscript, particularly when viewed in conjunction with the forecasts for Turkey in the revised version, strongly suggest that the currently proposed scheme with the kernel function, the hyperparameter ranges, and the month partitioning will yield unbiased and accurate NGD predictions. There is no data leakage or "selection bias."
